# The Potential and Challenges of Digital Well-Being Interventions: Positive Technology Research and Design in Light of the Bitter-Sweet Ambivalence of Change

**DOI:** 10.3389/fpsyg.2018.00331

**Published:** 2018-03-13

**Authors:** Sarah Diefenbach

**Affiliations:** Department of Psychology, Ludwig-Maximilians University, Munich, Germany

**Keywords:** positive technology, technology design, well-being interventions, user experience, bitter-sweet ambivalence, positive change strategies

## Abstract

Along with the dissemination of technical assistance in nearly every part of life, there has been growing interest in the potential of technology to support well-being and human flourishing. “Positive technology” thereby takes the responsible role of a “digital coach,” supporting people in achieving personal goals and behavior change. The design of such technology requires knowledge of different disciplines such as psychology, design and human-computer interaction. However, possible synergies are not yet used to full effect, and it needs common frameworks to support a more deliberate design of the “therapeutic interaction” mediated through technology. For positive technology design, positive psychology, and resource oriented approaches appear as particularly promising starting point. Besides a general fit of the basic theoretical conceptions of human change, many elements of established interventions could possibly be transferred to technology design. However, besides the power of focusing on the positive, another psychological aspect to consider are the bitter components inherent to change, such as the confrontation with a negative status quo, threat of self-esteem, and the effort required. The present research discusses the general potential and challenges within positive technology design from an interdisciplinary perspective with theoretical and practical contributions. Based on the bitter-sweet ambivalence of change as present in many psychological approaches of motivation and behavior change, the bitter-sweet continuum serves as a proxy for the mixed emotions and cognitions related to change. An empirical investigation of those factors among 177 users of self-improvement technologies provides initial support for the usefulness of the bitter-sweet perspective in understanding change dynamics. In a next step, the bitter-sweet concept is transformed into different design strategies to support positive change. The present article aims to deepen the discussion about the responsible role of technology as a well-being enhancement tool and to provide a fruitful frame for different disciplines involved in positive technology. Two aspects are highlighted: First, investigating well-being technology as a form of “therapeutic interaction,” focusing on the need for sensible design solutions in the emerging dialogue between technology and user. Second, a stronger consideration of the bitter-sweet ambivalence of change, utilizing (positive) psychology interventions to full effect.

## Introduction

Nowadays, technology provides assistance in nearly every part of life. Besides being a tool for practical tasks, an important channel to fulfill psychological needs such as popularity and relatedness, technology also functions as a medium to support physiological and psychological health and personal self-improvement. Under umbrella terms such as “positive technology” or “positive computing,” research and design explores technology for well-being and human potential (e.g., Sander, 2011; Botella et al., 2012; Calvo and Peters, 2012, 2014; Riva et al., 2016b, 2017). More specifically, the positive technology approach aims at combining the objectives of positive psychology with technology design (Botella et al., 2012; Riva et al., 2016b). The positive computing approach defines a similar aim, i.e., “the study and development of technologies designed to support well-being, wisdom, and human potential” (Calvo and Peters, 2012, p. 29). Beyond this and more generally it argues for the “the inclusion of well-being and wisdom into the experience design of all technologies,” suggesting that “even companies like Facebook and Apple should be evaluating how their products affect wisdom and well-being as part of the iterative design cycle” (Calvo and Peters, 2012, p. 29). Other approaches come from a more clinical perspectives and use technology as a new medium for therapy and health care. Along with this, e-health and behavioral intervention technologies have become a popular and promising approach among health care providers (see Free et al., 2013; Mohr et al., 2013 for recent reviews) and especially for workplace health promotion (e.g., Ebert et al., 2014). Also, in the field of consumer technology, self-optimization apps and gadgets have reached an enormous popularity, supporting self-improvement in various domains such as nutrition, meditation and mindfulness, time management and stress reduction, as well as sleeping habits, sports and physical activity (e.g., Conroy et al., 2014; Yang et al., 2015). Though relating to different domains and backgrounds, all these approaches share the endeavor to support positive change and self-improvement in their users and see technology as a valuable means. In this vein, the term positive technology is here used in a broader sense, relating to different kinds of technologies with the aim to support positive change and self-improvement, and not only explicitly positive psychology based ones.

While assigning technology the role of a personal coach or psychotherapist, supporting their users in personal development or even to counteract mood disorders (e.g., Braun et al., 2016), technology design assumes high responsibility. Designing for a positive human experience and well-being is a complex task (e.g., Desmet and Hassenzahl, 2012; Desmet and Pohlmeyer, 2013; Pohlmeyer, 2013), and possibly even more in the sensible domain of behavior change which asks for a careful consideration of psychological factors (e.g., Hassenzahl and Laschke, 2014). As highlighted in the present research, the support of behavior change must also consider the typically related bitter-sweet experience, consisting of mixed, positive and negative emotions. In the context of technology-supported change, this poses the question how to translate psychological knowledge into technology design, to find a representation that actually makes use of the interactive potential of technology, and to integrate knowledge from various disciplines. The field of positive technology obviously asks for a close collaboration between psychologists, designers, technology engineers, and others. However, until now, possible synergies between psychology and technology design are not used to full effect. Recent reviews in the context of health and well-being technologies (e.g., Free et al., 2013; Mohr et al., 2013; Conroy et al., 2014) suggest that literal interdisciplinarity is still missing.

On the one hand, psychology disregards the potential of interactive technology to affect people's routines beyond written instructions, e.g., through feedback, visualizations, and deliberate interaction design. Instead, psychology-based e-health interventions often transfer established trainings (e.g., cognitive behavioral therapy trainings) from offline mediums into videos and websites, so that technology is just an alternative way of distributing content. For example, a recent review on online positive interventions to promote well-being and resilience among adolescents (Baños et al., 2017) revealed a very limited number of utilized technologies, and strongly argues for a more intense utilization of smartphones, other mobile devices, sensors, and virtual/augmented reality technologies.

On the other hand, more technological advanced tools for behavior change often disregard the relevant psychological knowledge. A recent analysis of behavioral change techniques in mobile apps for physical activity found that the vast majority of commercial apps have not been evaluated using scientific methods and only few are explicitly grounded in theories of psychology or health behavior (Conroy et al., 2014). Overall, there is a strong focus on educational and cognitive aspects, but a disregard of the critical role of emotional and motivational factors for behavior change and long-term engagement (Conroy et al., 2014; Hollis et al., 2015; Yang et al., 2015). If the product does not “speak” to the user in the right way, change is sabotaged before it really started (see also Niess and Diefenbach, 2016). A survey among users of self-improvement technologies revealed a considerable ratio of users stopped using the technology before making significant progress, due to not feeling well supported (Diefenbach et al., 2016). Besides negative emotions while using the product (e.g., “made me constantly feeling guilty,” “was getting on my nerves”) such users complained about the product as being “too dominant,” “bossy,” “demanding,” or “stubborn.” In contrast, more satisfied users characterized the product as “motivating,” “gently reminding,” or “amusingly warning,” thereby hinting at the ambivalent character of inconvenient advices.

Altogether, the emerging dialogue between product and user and its emotional and motivational consequences appear as a central link between psychology and technology design for well-being. Though being aware that essentially just an algorithm is giving them advice, people accept technology as a coach and even develop a bond with it (e.g., Beun et al., 2016). Hence, parallel to the patient practitioner relationship as an important medium and vehicle of change in psychology (Ryan et al., 2008), and the product as an “argument in material form” in design (Redström, 2006), positive technology becomes a “medium of therapeutic interaction,” initiating a dialogue about change and ways to enhance well-being. The term “therapeutic” does not imply an exclusive focus on “serious” matters but emphasizes the responsibility related to any technology intervening in people's lives, behaviors, thoughts and feelings. More generally, if people see an aspect of themselves as a “project” to be improved, self-improvement technologies may be a way to support their wish for change. However, to be successful, it is of crucial importance to understand the user's experience of such technologies and to design these in a way that they trigger experiences and emotions that have proven as beneficial for positive change and achievement in psychological theory (e.g., control-value theory, Pekrun, 2006), see section Change as a Bitter-Sweet Experience for a detailed discussion.

To support this endeavor and synergies between disciplines, an important question is how to “translate” concepts from one discipline to another, as for example, how to consider psychological perspectives in interaction design. The present research provides one possible starting point for the translation of psychological knowledge about motivation and behavior change into the design of positive technology. More specifically, it focuses on the explicit consideration of the bitter-sweet ambivalence of change, as defined by the fact that any wish for change stems from the view that something is not ideal yet, which is basically a challenging, “bitter” experience. Regarding technology design, this poses the question how interactive technology could take a helpful “therapeutic attitude,” what would be an appropriate way to “speak” to the user and how this might be systematically realized through particular design elements that take the bitter and sweet components into account.

While many existing design approaches already utilize psychological theory to support positive user experience (e.g., Desmet and Hassenzahl, 2012; Desmet and Pohlmeyer, 2013; Hassenzahl et al., 2013), the present research advances these by a focus on the “bitter-sweet ambivalence” inherent to any wish for change and possibly related positive and negative emotions. While experiencing progress toward a goal is attractive, committing to a goal also comprises the confrontation with current deficits and the risk of failure. As already discussed in the approach of “frictional feedback” as a design strategy for behavior change, breaking up routines never comes without friction (Hassenzahl and Laschke, 2014; Laschke et al., 2015). Changing one's routines is always an effort and a challenging situation from a psychological and design perspective.

The following paragraphs summarize the general strength of positive psychology and resource-oriented approaches in the context of technology-mediated self-improvement as well as possible advancements of such approaches by an explicit consideration of the bitter and sweet aspects of personal change. Based on a working model, a preliminary empirical study explored the relevance of both kinds of change factors among users of self-improvement technologies. As a next step, three general strategies/starting points for the support of positive change and possible realizations through technology design are discussed. In sum, the motivation of the present paper is to provide a psychologically founded but not overly complex perspective on behavior change and hopefully a working ground for the area of positive technology, especially for interdisciplinary research and practice.

## Positive psychology interventions as a starting point for technology-mediated change

### Potential

In many respects, the core assumptions of humanistic psychology, positive interventions (e.g., Seligman et al., 2006; Biswas-Diener, 2010; Parks and Biswas-Diener, 2013; Vella-Brodrick, 2013) and resource-oriented approaches such as solution-focused coaching (Greene and Grant, 2003; Bamberger, 2011) go hand-in-hand with the idea of technology as a coach for personal change (see also Diefenbach, 2017 for a detailed overview).

First, there is the general belief in people's will and potential for personal growth and the client-as-expert view. Change is conceptualized as a function of autonomous motivation (e.g., Ryan et al., 2008), and being built on the utilization and revelation of the client's individual resources (e.g., Bamberger, 2011). The coach/therapist is considered an agent of change and moderator of development (Hermer, 1996), an assistant for self-management (Kanfer et al., 2006) or a supervisor of interaction with the outside world (Schmidt, 1996). In order to activate the client's potential for change, it just requires the right triggers and questions. This view makes it conceivable that a digital coach could trigger some of this potential as well. In contrast, within a psychoanalytic line of thinking—emphasizing the therapist's personal expertise and interpretation of the patient's reports, and phenomena such as transference and countertransference in therapist-patient communication—it would be hardly conceivable for technology to slip into the role of a coach or therapist.

Second, positive, resource-oriented approaches are indication independent, that is, they are not asking for the origin or development of problems but for solutions for the future. More important than what has been in the past are visions about a possible future. In fact, it is even argued that a focus on the problem, keeping thoughts turning in paralyzing circles, can often prevent rather than initiate positive change, which de Shazer et al. (de Shazer et al., 1986; de Shazer and Dolan, 2012) call “problem trance” or “problem hypnosis.” In contrast, new perspectives and imaginations, triggered through unusual and inspiring questions or exercises such as role plays, are appreciated as a playground to experience how it could be. This future-oriented view makes it much easier for technology to set helpful triggers, than if a full analysis of reasons, as in the past, was needed.

Third, the typical “toolset” of positive approaches, i.e., systematic sets of questions, framings, and reflections on goals and solutions that have proven promising triggers to individual solutions (e.g., Greene and Grant, 2003; Gamber, 2011), is transferable to technical representations. Many of these techniques, such as positive framing, systematic questioning, coaching as an invitation to face the challenges of the future, reflections through role plays, sculpture techniques, scaling questions, visualizations and working with metaphors and images could possibly be translated into online interventions, apps or even gameful approaches. Furthermore, technology could provide an advanced representations of such techniques beyond face-to-face coaching, e.g., by vividly showing “problem constellations” from different angles (visualizations) or making the effort for reaching gradual goals tangible through touch parameters representing psychological effort (section Strategies for Positive Change: Application of the Bitter-Sweet Concept in Psychological Interventions and Technology Design, strategies for positive change, discusses some of these ideas in more detail). A particular interesting potential is provided by augmented/virtual reality technologies, which are already used to promote positive change in different areas of behavioral health. Besides providing a controlled setting to develop and exercise new skills, such technologies are particularly useful to generate the feelings of personal efficacy and self-reflectiveness required for change (see Riva et al., 2016a for a recent review).

Finally, positive approaches put a high value on everyday practicing and the integration of positive activities in daily routines. Here, interactive technologies such as smartphone provide an easy channel to transfer positive interventions into existing routines, and support the practicing of new routines through memory functions etc. Given that the smartphone is already a daily companion for many, it offers a playful and lightweight way of reflection on personal strengths and potentials for change.

All the aspects discussed above provide generally good conditions for the integration of positive psychological approaches in technology design. However, in addition, the positive focus within the support of individual change also comes with particular challenges and limitations.

### Challenges and limitations

Per definition, the field of positive psychology at the subjective level is primarily concerned with the positive and valued subjective experiences, including “well-being, contentment, and satisfaction (in the past); hope and optimism (for the future); and flow and happiness (in the present)” (Seligman and Csikszentmihalyi, 2014, p. 280). However, this does not necessarily imply to trigger exclusively positive emotions as an ultimate goal. For example, considering the development of positivity in a long term perspective, one may ask “how much delayed gratification is necessary to increase the chances of long-term well-being?” (Seligman and Csikszentmihalyi, 2014, p. 293). Besides, already in a short term perspective, it may not be evident whether positive-focused interventions will actually have positive effect for the individual. In sum, one central challenge within positive psychology interventions seems to find the right level of positivity to support positive change in a given situation, particularly if transferred by technology. In the present context, the question of the right level of positivity is particularly relevant in two regards.

At first, it refers to finding appropriate forms for rewards and positive therapeutic reinforcement, to keep positive framing in a sensible range. Many people seem to like the “motivational quotes” of the running app Runtastic such as “I am not here to be average, I am here to be awesome!,” “Be the type of person you want to meet.” However, the same quotes can appear inappropriate, e.g., when the “personal journey to weight loss” turns out as a terrible failure. If the same “encouraging” comments follow each behavior, one's actions become at some point meaningless. While a human coach can more sensibly react to individual situations, technology has a more difficult job to detect what motivation one actually needs to flourish. Positivity is surely a good starting point, but still, the right dosage is needed.

Second, the sole focus on the positive can even be dysfunctional; positive psychology interventions can “backfire” and positive goals will reveal a “dark side” (Biswas-Diener, 2010, p. 66). The imagined ideal self becomes a source of frustration instead of motivation, and evokes anxiety rather than hope and inspiration. Contrasting the “real you” against the “ideal you” can give crucial feedback for the personal change process and illuminates areas for growth, but at the same time it can cause people to feel dejected instead of inspired (Biswas-Diener, 2010, p. 47). In general, interventions for positive change can have unintended negative side effects on different levels—also discussed as “persuasive backfiring” in the context of persuasive technology (Stibe and Cugelman, 2016). Though many supportive strategies can make change “sweeter” there are still “bitter” components, which one needs to acknowledge when developing the most helpful strategies for positive change. Again, a sensible consideration of bitter and sweet factors seems especially relevant in the context of technology-mediated behavior change, where no human coach can intervene in the critical moment.

While the low threshold related to “seeking advice” from interactive technology generally can be seen as an advantage—a lightweight possibility for self-reflection, a little tool one can test in a playful manner, not necessarily associated with a confession of needing help—technology also provides a low barrier to abandon the whole process. Trying to change may result in frustration or the simple insight that change is actually hard work. It is only a small step to delete “the damn app” and to get rid of the frustration. Here, the experienced or imagined bitterness may be a reason to stop or even not start projects of change. As already concluded by Kanis and Brinkman (2009, p. 127), “there is clearly an opportunity to employ technology for positive change, but how this can be achieved is more difficult to determine.”

The present article suggests that one critical aspect for the successful design of positive change technologies is the explicit consideration of the “bitter components” of change, i.e., the potential hurdles and barriers that might arise when trying to implement positive goals in daily life and the nearly inevitable confrontations with shortcomings on the way to change. To have full effect, positive technology must not stay focused on the positive alone but also support people in dealing with challenging situations (Sander, 2011).

## Change as a bitter-sweet experience

The intention to change is always a bitter-sweet experience, typically accompanied by mixed emotions and a combination of positive and negative feelings. Considering a wish for personal change as a gap between the actual and ideal self, this naturally includes a feeling of inadequacy, often coming with dejection-related emotions such as disappointment, dissatisfaction or sadness (Higgins, 1987). Hence, on the sweet side of change there are the attractive goals, the positive belief of being able to achieve such goals, the vision of becoming the person he or she wants to be, and if quick progress is achieved- the encouraging feedback. On the bitter side, there are the confrontations with current deficits, the risk of failure and self-blaming, the threats to self-esteem and the necessary effort to approach one's ideals. In the following, the bitter-sweet perspective forms a working concept for different facets of conflicting or antagonizing forces. This view is in parallel to many psychological approaches and models in the area of motivation and behavior change. While not being exhaustive, the aim of the present compilation is to point out the tension bitter and sweet components, and to exemplify representatives of the bitter-sweet ambivalence in change and progress with regards to existing theory.

An example of bitter-sweet conceptions in theory of change is provided by the intentional change theory (ICT, Boyatzis, 2006), and the role of positive and negative emotional attractors in personal change (e.g., Howard, 2015). While the positive emotional attractor (PEA) comprises personal hopes, dreams, possibilities, strengths, optimism and self-set goals that make up our ideal self, the negative emotional attractor (NEA) comprises the present reality, fears, problems, shortfalls, pessimism and improvement goals related to our real self. Also, the famous flow theory (Csikszentmihalyi, 1990) emphasizes that positive experience and progress results from challenge (rather bitter) and skill (rather sweet) at the same time. Bitter and sweet components may also be related to the formation of concrete action plans, as suggested in many theories on self-regulation and behavior change (e.g., Gollwitzer, 1999; Schwarzer, 2008). Gollwitzer (1999) recommends the formulation of clear implementation intentions, that is, concrete if-then plans to implement behavior change in daily life. On the sweet end, such action plans offer the opportunity to change, or a concrete way to goal attainment that “just has to be followed.” On the bitter end, the concreteness of such action leaves no space for escape and excuses. Even if self-prescribed—when confronted with the inevitable call for action—the clear demand to change may be experienced as restriction of autonomy and result in reactancy (Brehm, 1966).

Another example highlighting a form of bitter-sweet interplay is the optimal margin of illusion hypothesis (Baumeister, 1989). It associates optimal psychological functioning with a slight-to-moderate degree of distortion in one's perception of oneself, and suggests a balance between a realistic (rather bitter—if not being perfect) and an optimistic, positive view of oneself (sweet). Though the optimal margin hypothesis has also been challenged (e.g., Brookings and Serratelli, 2006), it might still form a helpful metaphor when thinking about design strategies for behavior change. To keep people in the optimal margin, a coach (human or digital) would confirm people in their positive view, thereby utilizing first small changes as a resource for power and further change, but without becoming overly optimistic and losing out of sight what still needs to be done.

Similarly, theories of persuasion and message design include bitter-sweet dimensions as well. For example, the Persuasive Health Message (PHM) Framework (Witte, 1995, p. 146) suggests that in order to motivate change, a threat message is needed, to make the audience feel susceptible to a severe threat (bitter) as well as an efficacy message, to convince individuals that they are able to perform the recommended response (sweet). Negative emotions such as fear can be an inhibitor but also a motivator (Witte, 1998), so that, in the right dosage, the experience of a bitter component might support transformation. While originally the PHM framework refers to health-message design for public campaigns, positive technology designs health and well-being messages in the form of technology.

Finally, interesting parallels can be drawn to research on academic learning and achievement, especially the control-value theory (CVT) introduced by Pekrun et al. (e.g., Pekrun, 1992, 2006; Pekrun et al., 2006). In parallel with the present consideration of bitter and sweet components of behavior change, CVT provides a dedicated description of the combined functions of positive and negative emotions in self-regulation and learning. More specifically it describes the interplay of positive outcome-related (e.g., anticipatory joy, hope) and activity-related emotions (e.g., enjoyment) as well as negative outcome-related (e.g., anxiety, sadness) and activity-related (e.g., anger) achievement emotions, and their relations to control and value appraisals. An important aspect related to the present idea of the bitter-sweet ambivalence of behavior change, and the explicit acknowledgment and utilization of bitter change factors, is the view of negative emotions as not necessarily being detrimental to self-regulation and learning and positive emotions not necessarily being beneficial. CVT acknowledges that positive achievement emotions do not always exert positive effects, and negative achievement emotions do not always produce negative effects (Pekrun, 2006, p. 327). Besides the valence of emotions, also their activating potential does play a role. Pekrun (2006 p. 326) concludes that for most task conditions “the effects can be assumed to be beneficial for activating positive emotions like enjoyment of learning, detrimental for deactivating negative emotions like boredom and hopelessness, and more ambivalent for both deactivating positive emotions such as relaxation, and activating negative emotions such as anxiety of such emotions.” Another interesting perspective in CVT is the emphasis on habitualized emotions. As Pekrun (2006) explains, based on procedural schemes, situational perceptions alone can be sufficient to induce particular habitualized emotions and names the example of a student's habitualized anxiety upon entering the classroom. Obviously, such habitualized emotional reactions can be a serious barrier to positive behavior change. However, interactive technologies could be a chance to break up such pattern and provide a new frame and a chance to develop, other, more beneficial emotional reactions. Pekrun (2006 p. 324) lays out that, “whenever the situation changes [however], appraisals come into play again, and changes of appraisals may change habitualized emotions.” In sum, the assumptions of CVT thus provide a useful frame for the present idea of the bitter-sweet ambivalence in context of interactive technologies supporting behavior change.

Note that for some of the above mentioned concepts and theories the bitter-sweet parallel refers to an explicit negative-positive differentiation and the interplay of both types of emotions (e.g., Boyatzis, 2006; Pekrun, 2006). In other cases, the negative–positive interplay is less explicitly formulated (e.g., threat vs. efficacy messages, Witte, 1995) or results from the present interpretation of different concepts in combination and potential arising conflicts. This, for example, refers to the double-sided psychological effects of concrete behavior plans, i.e., the positive/sweet activating power of implementation intentions (Gollwitzer, 1999) in contrast to the negative/bitter deactivating power of reactancy (Brehm, 1966). In order to cover such manifold perspectives and provide room for a broad interpretation, it was a deliberate decision not to limit the concept to a clear cut negative–positive differentiation, but to use the more colloquial and ambiguous term bitter-sweet. This also refers to the interdisciplinary perspective, aiming to provide starting points for various disciplines concerned with the research and design of positive technology, instead of restricting the scope to particular psychological concepts.

### A working concept: bitter vs. sweet components and crucial stages along the change process

**Figure 1:**
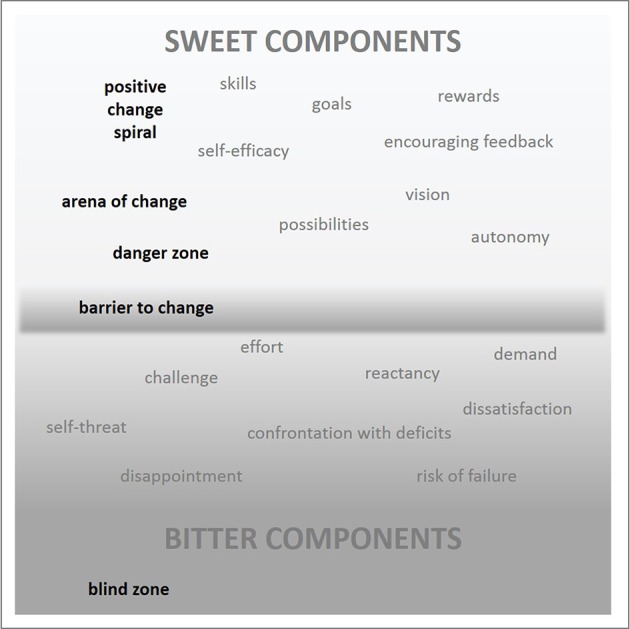
A working concept: change as a bitter-sweet experience.

In simplified terms, bitter-sweet experiences of change can be envisioned along a continuum (see Figure 1). The bitter component is inevitable and needed to a certain degree. A wish for change often originates from the bitter experience of a discrepancy between real and ideal self. However, the interplay between bitter and sweet components is critical to change success. If the bitter component is too dominant, no change takes place. Even if one becomes aware of a discrepancy between real and ideal self, and catches a glimpse of one's own wish for change, an escape back into the *blind zone* is possible. The blind zone is considered to be a comfort zone, where the confrontation with deficits becomes “invisible” again, e.g., through denial or the avoidance of confrontation with discrepancy between real and ideal self. Thus, a first critical stage for change is crossing the barrier from where the degree of bitterness seems “do-able,” and one can commit oneself to a wish for change. This is the starting point for the actual *arena of change*. Having arrived at this point, the primary challenge lies in sustained engagement in change. Only if the first steps of change are a rewarding experience, one will enter the *spiral of positive change*, where change becomes increasingly sweeter and further fuels the motivation to change. Such kind of positive escalation corresponds to the concept of *early reactivity*, i.e., a rapid increase in positive emotions after starting an intervention, which has been acknowledged as vital factor for successful positive psychology interventions (e.g., Cohn and Fredrickson, 2010; Proyer et al., 2015). Increasing competency, feelings of self-efficacy and approaching one's ideal, and at the same time, reduced effort, disappointment and self-threat, could all add to the experience of positive reinforcement. In contrast, if one experiences his or her first steps of change as ineffective/failure, one will fall back into the *danger zone* around the critical change barrier. As such, the perceived bitterness of change can become even stronger than before—failing after having commitment to change tastes more bitter than it did before having tried. For the sake of self-protection and dissonance reduction (Festinger, 1962), one may declare the original goal as “wrong” and deny one's original ideals. A lack of early reactivity thus forms one of the most severe dangers in the process of change. If a chosen intervention path for change does not have any positive effects, it is suggested to acknowledge this and search for another, more promising alternative, rather than prevailing in the experience of ineffectiveness (see also Proyer et al., 2015).

### Key assumptions and possible applications of the bitter-sweet concept

In sum, the present conceptualization of change assumes the following: Change always includes bitter and sweet components. Setting goals includes the risk for failure and self-threat, and change (i.e., breaking up routines) is struggling *per se*. Even if the ultimate goal is to enjoy the sweet side (e.g., finally experiencing sports as an intrinsically motivating activity that actually feels good—and not as a duty), the bitter side should not be neglected, but could be even utilized as a vehicle of change (the power of dissatisfaction). However, bitter and sweet components must be in a bearable ratio—if the bitter is too dominant, one might rather deny the wish to change. Thus, instead of solely focusing on the positive, and thereby risking unforeseen consequences and backfire effects, strategies for change should actively consider its bitter-sweet character. From a design perspective, this could imply thinking about factors evoking rather bitter/negative (e.g., confrontation with deficits) or sweet/positive emotions (e.g., encouragement).

The present perspective on change, whereby the bitter-sweet concept serves as a proxy for the ambivalence of change (Figure 1), allows to delineate user's current position within a change process, relevant forces and mechanisms, the most needed kind of support, and related design strategies. The core intention is to create a representation of the inherent ambivalence of change, and to make this graspable within the envisioning of strategies and technology design for positive change.

The bitter-sweet concept can provide a common frame for different disciplines involved in the field of positive technology, i.e., relations to psychological theory and mechanisms, and at the same time concrete starting points for design and the utilization of technology to support change. As recently stated by Gaggioli et al. (2017, p. 496) when discussing a research agenda for the field of positive technologies, “Finding the right way of communicating ideas, in a way that resonates with distinct academic communities is not trivial and is maybe in itself the first important topic of any research agenda.” In this vein, the present research aims to provide a simplified but psychologically-oriented view on change processes and related user experiences, with potential for different communities. Note, however, that the suggested working concept does not claim to represent distinct psychological processes, nor does it make hypotheses about specific interrelations of the different bitter and sweet components. While this might be a goal for future research, the present paper aims at a first exploration of the general usefulness and viability of the concept.

The next section presents a preliminary empirical investigation of the bitter-sweet conceptualization among 177 users of self-improvement technologies. The section thereafter explores different design strategies for positive change along the bitter-sweet continuum.

## Preliminary empirical investigation: bitter and sweet change factors in self-improvement technologies

A user study within the context of self-improvement technologies served as a first empirical exploration of the potential relevance of bitter and sweet factors in processes of personal change. The study focused on four change factors in particular, i.e., *confrontation with deficits* and *demand* on the bitter end as well as *autonomy* and *encouraging feedback* on the sweet end. *Confrontation with deficits* addresses the confrontation with deficits, such as visualizations of the status quo and gaps between ideals and reality. *Demand* comprises the clear and definite call to change, including concrete instructions, which, however, might also be experienced as negative and cause reactancy (Brehm, 1966). Both factors, *confrontation with deficits* and *demand*, thus address change from the bitter end: change is initiated through the confrontation with a negative state that is declared as not acceptable. This also parallels the “notion of activity trackers as “deficit” technologies, to which people turn when they are afraid of failing” (Gouveia et al., 2015, p. 1309). In other words, the motivation to change is to prevent the negative. In contrast, *autonomy* and *encouraging feedback* refer to positive attractors of change, namely, actively influencing conditions for progress, experiencing oneself as creator of change, and confirmation of success. *Autonomy* refers to the degree a product supports autonomous decisions regarding the way of change and goal attainment*. Encouraging feedback* refers to positive responses to the user's actions; for example, by means of rewards for first small steps in the change process.

### Methods

One hundred and seventy seven users of self-improvement technologies answered a survey and provided reports on their personal change process as well as ratings on the used technology by various measures. As an incentive, three 15-euro gift vouchers were raffled among all participants. The study was conducted online via unipark (unipark.com) and a convenience sample (127 female, mean age 31 years, min = 18, max = 65) was recruited via various university mailing lists and social media groups. Though we do not have exact data about the popularity of self-improvement technologies among women and man, the skewed female sample is in line with other studies exploring self-improvement technologies and similar approaches within convenience samples (e.g., Yang et al., 2015; Chittaro and Vianello, 2016). Similarly, a recent survey about the share of smartphone owners using fitness apps in Germany also showed a higher ratio of female users (Statista, 2015).

The study was carried out in accordance with the APA ethical standards and the German Psychological Society's (DGPs) ethical guidelines (2016). According to the DGP's ethics commission, an institutional research board's ethical approval is only required if any funding is subject to such an ethical review. No such requirements were present for this study. Participation in the study was voluntary. Participants were assured of anonymity and confidentiality. Participants were informed about the purpose of the research, expected duration, procedures, the study incentive, their right to cancel and withdraw their consent for participation at any time during the study, and a contact person for any questions or concerns regarding the study. All subjects gave written informed consent in accordance with the Declaration of Helsinki.

After having provided their consent, participants were asked to name a self-improvement technology which they are currently using, and describe their product and experience by various measures.

Each of the above described bitter and sweet change factors (*confrontation with deficits, demand, autonomy, encouraging feedback*) was assessed with three items (as listed in the Appendix, see also Mehner, 2016). Sample items are “The product …” “makes me realize that have not yet reached my ideal” for the factor *confrontation with deficits* or “provides positive feedback” for the factor *encouraging feedback*. The item development was inspired by relevant conceptualizations in psychology, coaching, persuasive technology design and health research. More specifically, the items to assess *confrontation with deficits*, were oriented on the transtheoretical model of health behavior change (Prochaska and Velicer, 1997), which emphasizes raising awareness of a problem and the need to change in the phase of contemplation before entering the phase of preparation, in which people are intending to take action in the immediate future, as well as the principles of motivational interviewing (Miller and Rollnick, 2012). One of these principles, namely, “develop discrepancy” suggests that change is motivated by highlighting the discrepancy between patient's perceived goals and values vs. current behavior. In line with the prominent three-component description of psychological attitude (e.g., Eagly and Chaiken, 1993), the items covered affective (… confronts me with my dissatisfaction), behavioral (… that I haven't done enough yet) and cognitive (… makes me realize that I have not yet reached my ideal state) aspects of deficit confrontation.

The assessment of *demand* was oriented on the taxonomy of behavior change techniques by Michie et al. (2011) and the behavior model for persuasive design by Fogg (2009). One of the behavior change techniques described by Michie et al. (2011) is “action planning.” Compared to other more abstract techniques such as goal setting, action planning asks for detailed planning of what the person will do including, as a minimum, when, in which situation and/or where to act. This clear link between specific situational cues and behavioral responses does hardly provide room for excuses or avoid getting active any longer. Also in Fogg's model (Fogg, 2009) there is an emphasis on signal triggers that serve as a reminder to start the intended behavior. This relentless attitude and the initiation of change through particular (situational) triggers was represented in items such as “The product … does not accept that I put off getting active any longer” or “… definitely reminds me to start the planned behavior change.”

The user's perceived *autonomy* within the process of change and goal attainment was assessed in parallel to items on autonomy and self-determination in the working domain and the research by Spreitzer (1995) on psychological empowerment in the workplace. Sample items from the self-determination scale by Spreitzer (1995) are “I have considerable opportunity for independence and freedom in how I do my job” or “I can decide on my own how to go about doing my work.” In the present context, this was translated into items such as “The product … provides considerable freedom about how to reach my goals” or “… leaves it up to me how to design the change process.”

Finally, the items to assess *encouraging feedback* were derived from typical formulations used in the context of solution-focused coaching (Greene and Grant, 2003; Bamberger, 2011). Solution-focused coaching shift the client's focus on resources, improvement, first signs of reaching the goal and the appreciation of already taken steps toward the envisioned future. The coach typically expresses encouraging feedback such as “I am deeply impressed how you managed this difficult situation” or, to activate the client's resources, “Which resources did help you to initiate this first step?” In the context of self-improvement technologies, this attitude was reflected in items such as “The product … … praises me for my actions” or “… acknowledges small steps on the way to self-improvement.”

For each item, participants indicated their degree of agreement on a seven-point-scale (1 = not at all, 7 = completely). Scale values were computed by averaging the corresponding items; the internal scale consistency was satisfactory (Cronbachs alpha: *confrontation with deficits*: 0.71, *demand* 0.76, *autonomy* 0.79, *encouraging feedback* 0.83). A principal component analysis (varimax rotation, 72% explained variance) with four components to be extracted revealed a satisfactory solution with no loadings larger than 0.30 on other components, expect for one cross-loading between *demand* and *confrontation with deficits* (0.54), and one between *encouraging feedback* and *autonomy* (0.32), see Appendix for the matrix of factor loadings.

Besides ratings on the bitter and sweet factors, participants further rated their product and experience of change by the following measures: global product evaluation (1 = bad, 7 = good), positive and negative affect (1 = not at all, 7 = extremely) and point of time within the change process (1 = early stage, 7 = advanced stage). Change success was assessed with three items (Cronbachs alpha 0.89, namely, “I have reached my goals with the help of the self-improvement technology,” “I realize I already made progress toward my goals” and “The self-improvement technology supported me in becoming who I want to be.” Again, participants indicated their degree of agreement on a seven-point-scale (1 = not at all, 7 = completely).

### Findings and discussion

The sample of self-improvement technologies consisted of smartphone apps and gadgets from various domains. The most commonly rated products were fitness apps, nutrition apps, language learning apps, and fitness gadgets such as step counter wristbands. Table 1 lists frequencies and sample products/apps for the different product categories.

**Table 1:** Self-improvement technologies under study.

**Product category**	**Frequency**	**Sample products/apps**
Fitness apps	53 (30%)	Runtastic, Freeletics, 7 Min Workout
Nutrition apps	34 (19%)	Weight Watchers App, MyFitnessPal, Lifesum
Language apps	32 (18%)	Babbel, Busuu, Duolingo, Obenkyo
Fitness gadgets	20 (11%)	Polar M400 running watch, Mi Band fitness & sleep tracker
Health apps	16 (9%)	Health, S Health, Global Corporate Challenge
Relaxation apps	6 (3%)	7Mind, Provider Resilience
Other	16 (9%)	card2brain, NeuroNation, Memrise

The pattern of correlation revealed that the sweet factors, *autonomy* and e*ncouraging feedback*, are more relevant for a positive product evaluation (see Table 2, row 7–8) than the bitter factors, *confrontation with deficits* and *demand* (Table 2, row 4–5). However, both kinds of factors, bitter and sweet, were positively correlated to change success. Even though the bitter factors may not lead to “liking” the product, participants are well aware of their impact on personal change. A stepwise linear regression, using change success as the criterion and all four change factors and interaction terms as predictors revealed the interaction term *demand* × *autonomy* as most relevant predictor (*R* = 0.59, *R*^2^ adjusted = 0.31, β = 0.56, *p* < 0.001). This suggests that the process of change is especially successful if the product provides a clear demand to action, but at the same time, offers some degree of autonomy in the implementation. Beyond this, *encouraging feedback* was the only further predictor that could explain significant additional variance (*R* = 0.52, *R*^2^ adjusted = 0.37, β = 0.29, *p* < 0.001).

**Table 2:** Correlations between bitter/sweet factors of change, product evaluation, and change success (*N* = 177).

	**Product evaluation**	**Change success**
**BITTER FACTORS**		
Demand	0.17^*^	0.46^**^
Confrontation with deficits	0.07	0.30^**^
**SWEET FACTORS**		
Autonomy	0.34^**^	0.35^**^
Encouraging feedback	0.28^**^	0.46^**^

* *p < 0.05*,

** *p < 0.01*.

Another deciding factor for participant's product evaluation and experience, and the relevance of the bitter and sweet change factors, was the point of time within the change process. In general, with advanced stages of change, users felt more positive (*r* = 0.30, *p* < 0.001), less negative (*r* = −0.33, *p* < 0.001), reported a higher degree of change success (*r* = 0.44, *p* < 0.001) and rated to the product more positively (*r* = 0.30, *p* < 0.001). Moreover, a contrast of users in rather early stages (see Table 3, column 2–3) and rather advances stages (Table 3, column 4–5) by median split showed differences in the correlation pattern between bitter/sweet factors, product evaluations and successful change completion. While in the early phases, the sweet factors are more important for product evaluations and perceived change success; later on, the bitter factors become relatively more important.

**Table 3:** Correlations between bitter/sweet factors of change, product evaluation, and change success for early stages (*n* = 89) and advanced stages of change (*n* = 88).

	**Early stages of change**		**Advances stages of change**	
	**Product evaluation**	**Change success**	**Product evaluation**	**Change success**
**BITTER FACTORS**				
Demand	−0.04	0.40^**^	0.29^**^	0.44^**^
Confrontation with deficits	−0.06	0.23^*^	0.17	0.34^**^
**SWEET FACTORS**				
Autonomy	0.31^*^	0.28^**^	0.16	0.20
Encouraging feedback	0.29^**^	0.51^**^	0.24^*^	0.42^**^

* *p < 0.05*,

** *p < 0.01*.

A possible interpretation of this correlation pattern is the following: In the early stages of change, bitter factors of change are associated with negative experience and must remain in low levels to be acceptable. It takes some time for users to experience that some bitterness is actually helpful for reaching their personal goals, thereby adding to a positive product evaluation. Thus, in later stages of change, users may even acknowledge the motivation activated through bitter factors and hard words. Users learn that rewarding sweet experience results from personal efforts and bitter and sweet factors may go hand-in-hand. If one has experienced the positive change spiral at once, this serves as an additional attractor for ongoing engagement and occasional bitter experience becomes more bearable. Although failure and rebounds are still frustrating, the increasing ability to cope with bitter factors may become a rewarding experience itself.

This line of interpretation is also in parallel with the description of positive and negative feedback loops in relation to the above-mentioned (CVT) (Pekrun, 2006), referring to reciprocal causation effects between achievement emotions and appraisals in the context of learning and emerging dynamics over time. CVT posits two groups of appraisal as of specific relevance for achievement emotions (Pekrun, 2006 p. 317): (1) control appraisals, i.e., the subjective control over achievement activities and their outcomes (e.g., expectations that persistence at studying can be enacted, and that it will lead to success); and (2) value appraisals, i.e., the subjective values of these activities and outcomes (e.g., the perceived importance of success). As Pekrun (2006 p. 327) further points out control and value appraisals are assumed to be important determinants of emotions, but emotions can also reciprocally affect these appraisals. Such reciprocal causation can consist of positive feedback loops (e.g., enjoyment of learning and mastery at learning reinforcing each other) but also negative feedback loops (e.g., test anxiety inducing motivation to avoid failure, and resulting success reducing test anxiety). The dynamics of feedback loops can take place within varying time frames, from fractions of seconds, within learning episodes, or over days, weeks, and years (Pekrun, 2006 p. 327).

From the perspective of CVT, the present finding of an increasing importance of bitter factors over time could be related to a change in relevant appraisals over time. If users experience an increasing mastery to handle the challenges the technology asks for, this is an increase in control appraisals, here, being capable to actually take advantage of a product's “bitter” demand and confrontation with deficits. In other words, the user gains competency in making use of the potential of bitter factors, which as time goes by, actually do not seem so bitter anymore. Similarly to an experienced but strict sports coach, which may be advantageous for advanced sportsmen in later phases but totally demotivating for beginners in early phases of learning.

Nevertheless this is only one possible line of thinking why a gradually increasing degree of bitterness induced by the self-improvement technology could be desirable. Another limitation of the present findings is that the here applied measure of the point of time within the change process did not ask for the exact duration (e.g., in days, weeks, or months) but just captured a broad contrast of early vs. advanced stages. Future studies must thus substantiate these tendencies and provide a more complete picture, also including longitudinal research.

However, the revealed pattern already suggests that specific user needs may play a role in different stages of change processes. Based on the initial support for the general relevance of bitter and sweet factors in change in the user study, the next sections discuss how design strategies for positive change might address these factors. Note that while the user study underlined the general relevance of bitter and sweet for experienced change success, the following strategies are not inferred from the user study but rather depict general starting points to address the bitter-sweet concept in psychological interventions and how to integrate it in technology design. All strategies pick up existing psychological concepts or elements of established coaching techniques, however, future studies must explore their actual usefulness and applicability in the context of interactive technology.

## Strategies for positive change: application of the bitter-sweet concept in psychological interventions and technology design

**Figure 2:**
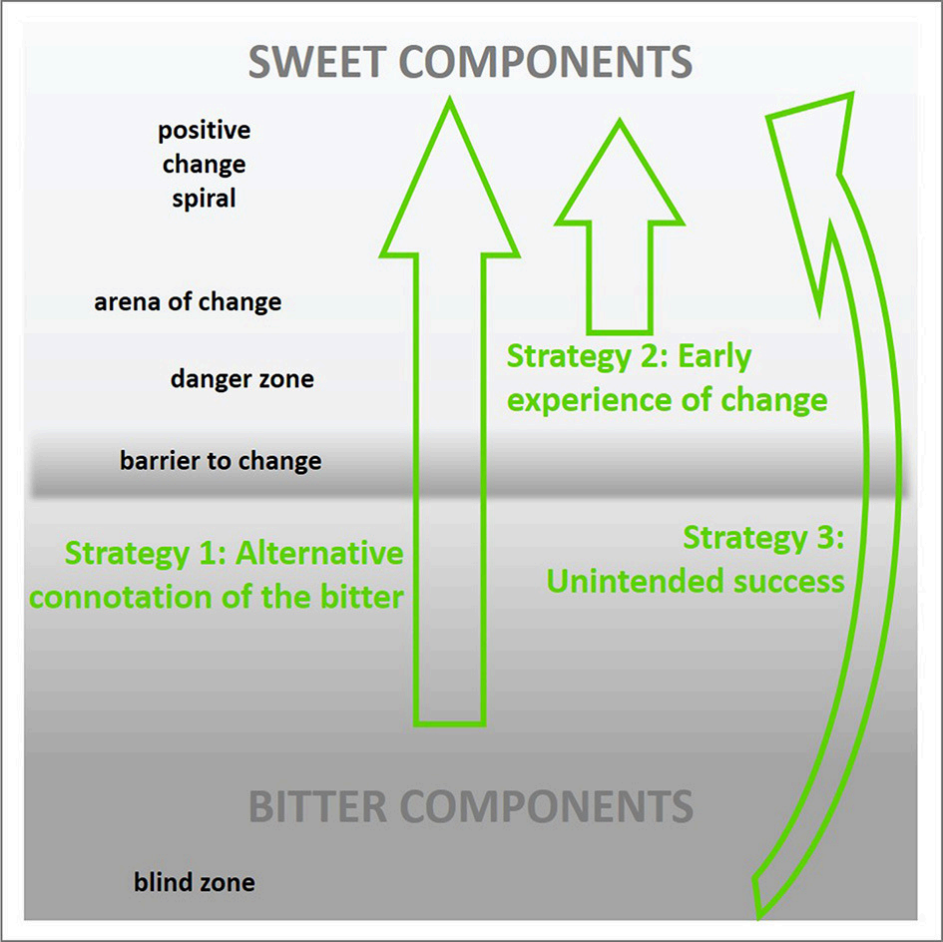
Different strategies for the utilization of bitter and sweet components in positive change interventions and design of interactive technology.

The notion of change along a bitter-sweet continuum provides a ground to conceptualize general strategies for positive change as well as their consideration in technology design (see Figure 2). This includes, for example, the positive connotation of bitter component, by making deficits appearing as a potential. Another example is represented by the support of self-enhancing processes in the positive change spiral, further strengthening the sweet components of change. Though taking different paths, the three strategies altogether aim at strengthening the chances for positive change by a helpful ratio between bitter and sweet, and preventing an escape back into the blind zone, where one denies one's wish to change, due to too much experienced “bitterness.” The following paragraphs draft these possibilities in more detail. For each strategy, a general introduction and relations to psychological concepts and coaching techniques is provided, followed by some suggestions how to address these aspects in interaction and technology design. Note, however, that the listed strategies are not meant to be exhaustive. The primary aim of the present collection is to highlight the different general options how to utilize the bitter and sweet in conceiving strategies for change—some primarily related to the bitter (e.g., alternative connotation), some primarily related to the sweet (e.g., early experience of change). Table 4 provides a summary of the different strategies, starting points along the bitter-sweet continuum, intended effects, related concepts from psychology and coaching, as well as possible realizations through technology.

**Table 4:** Three strategies for positive change.

**Strategy**	**Starting point**	**Intended effect**	**Related concepts/coaching techniques**	**Possible realization through interactive technology**
Alternative connotation of the bitter	Around the critical barrier, before full commitment to change	Transfer pure bitterness into a something sweeter, more energizing	Positive re-framing De-medicalization Tentative change Scaling questions A treasure hunt for own strength and resources	Labels, stories Visualizations Game-like interpretation of change processes Well-being treasure hunt
Early experience of change	Arena of change	Push into the loop of positive change Prevent drift back into the danger zone	Early reactivity Self-efficacy Re-activation of positive resources Confrontation with own success, activating further resources Acknowledging positive side effects Alternative perspectives Solution focus	Constant documentation of change Reminders, push-up notifications Visualizations, interaction attributes Feeling psychological weight and change in perspectives through gestural interaction
Unintended success	An (unplanned) jump from bitter to sweet, avoiding the barrier to change	Risk-free change No self-blame *Post-hoc* commitment	Ordeals Positive surprise about oneself Awareness of hidden skills, activation of positive resources Prevention of self-blame	Apps providing missions/tasks Hidden missions within general missions Retrospective reflection on success

### Strategy 1: alternative connotation of the bitter

A first strategy addresses situations around the critical barrier, before full commitment to change. The idea is to provide an alternative connotation of bitter components that transfer pure bitterness into a more positive and energizing construct, thereby still keeping the potential energy for change: the insight that “something needs to be done” in order to reach a positive goal. Such an alternative connotation of bitter components in the individual's reflections on change, related feelings and expectations and especially a reduction of self-threatening connotations, may enhance the odds for crossing the barrier to change. This idea is in parallel to typical re-framing interventions in the context of positive psychology and solution focused coaching (e.g., Greene and Grant, 2003). Instead of “fighting against being wrong” change becomes an act of “caring for oneself.” This approach also represents a form of de-medicalization (e.g., Broom and Woodward, 1996). Often, taking a medicine means being ill (self-threatening); thus, relabeling the medicine as an energy pill can turn a bitter situation in a sweeter one. Marketing professionals know very well that people feel better with using a “beauty serum” instead of “anti-wrinkle cream,” “pure products” instead of “anti-allergy products” or “fitness food” instead of “diet food.” Alike, it makes a difference whether an intervention is framed as “anti-stress training” or as “personal well-being training.” While the first term reminds one of being wrong (self-threatening) the second emphasizes the possibilities a change entails and evokes a desire to change in this way.

Interactive technology could create alternative, “sweeter” connotations of change in different kinds of manifestations. For example, when thinking about smartphone apps or online interventions, labels and stories embedding the intervention could trigger a sensible therapeutic frame. This could be, for example, a “well-being treasure hunt,” taking up a metaphor from solution-focused coaching: The client turns into a successful treasure hunter, hunting for his or her own strengths and resources. In this scenario, the coach only assists the client to seize the treasure and bring it to consciousness (e.g., Bamberger, 2011, p. 45). In an interactive game, the user could thus take the role of a treasure seeker or adventurer on an exploration tour. On a smaller level, positive framing could inspire the names of weekly missions, e.g., a nutrition app, which invites the user to a “fitness week” (sweet, emphasizing gains) instead of labeling the same thing as a “meat-free week” (bitter, emphasizing restrictions). In general, all kinds of game-like interpretations of change processes offer a great opportunity to play with one's own abilities in the form a noncommittal “test wise” change. By pushing considerations about self-threat and potential failures to the background the barrier to change becomes lower. Such playful, self-esteem neutral reflections or invitations for “tentative change” are a typical and very effective element from systemic therapy and solution-focused coaching (e.g., Greene and Grant, 2003). Also scaling questions (Bamberger, 2011) and other techniques which provide a supportive perspective on problems vs. progresses (i.e., the bitter-sweet ratio), could be easily realized, and even more, enhanced through visualizations in interactive technology.

### Strategy 2: early experience of change

A second strategy addresses the processes in the arena of change, after the barrier has been crossed and first trials have started. The aim is to push people into the loop of positive change and prevent a drift back into the danger zone, where they might give up. As discussed above, a central element in the arena of change is early reactivity, that is, a rapid perceivable effect of one's activities to change, confirming the general effectiveness of an intervention (Cohn and Fredrickson, 2010; Proyer et al., 2015). To give an everyday example: aching muscles after a first workout may signal “development in progress” and that one's activity actually had “some effect.” Positive comments by others such as “You look good today, somehow fitter,” may further support the experience of self-efficacy.

The foremost advantage of interactive technology to create such an early experience of change is the omnipresence of technology in daily life. For many, smartphones are constant companions. Unlike a human coach, smartphones may accompany the client through the hurdles of daily life and re-activate resources when needed. A common problem in classical face-to-face coaching is that the client actually shows progress, but does not recognize the already performed change to full extent. Here, technology could support a continuous documentation and appropriate expression of progress. In fact, the specific way that progress is documented can be crucial to its motivating power. For example, instead of hard, fixed numbers and physical metrics, progress could be expressed through a more appropriate, more flexible “currency.” An effort based time reduction from 25:30 min to 25:05 in a five kilometer running distance is not appropriately acknowledged by raw time data. Getting 100 progress points acknowledges this improvement considerably more. Similarly, in the field of nutrition: Keeping up the motivation after the first gains have been achieved and the curve of change becomes flatter is a difficult task. While losing the first kilos might be a relatively easy process, further progress is not reached with the same gradient. To prevent demotivation, visualizations and scores provided by technology could acknowledge the many parallel positive effects in other areas of healthy nutrition. Hence, every healthy day could be acknowledged as a day of value, by adding extra acknowledgments on different levels of well-being and long-term goals such as vitality, life-expectancy, or simply joy of life. Occasional backlashes on one level will become more bearable when set against progress on other levels. Inevitably, the user is confronted with what has already been achieved, activating one's skills and resources for further change.

**Figure 3:**
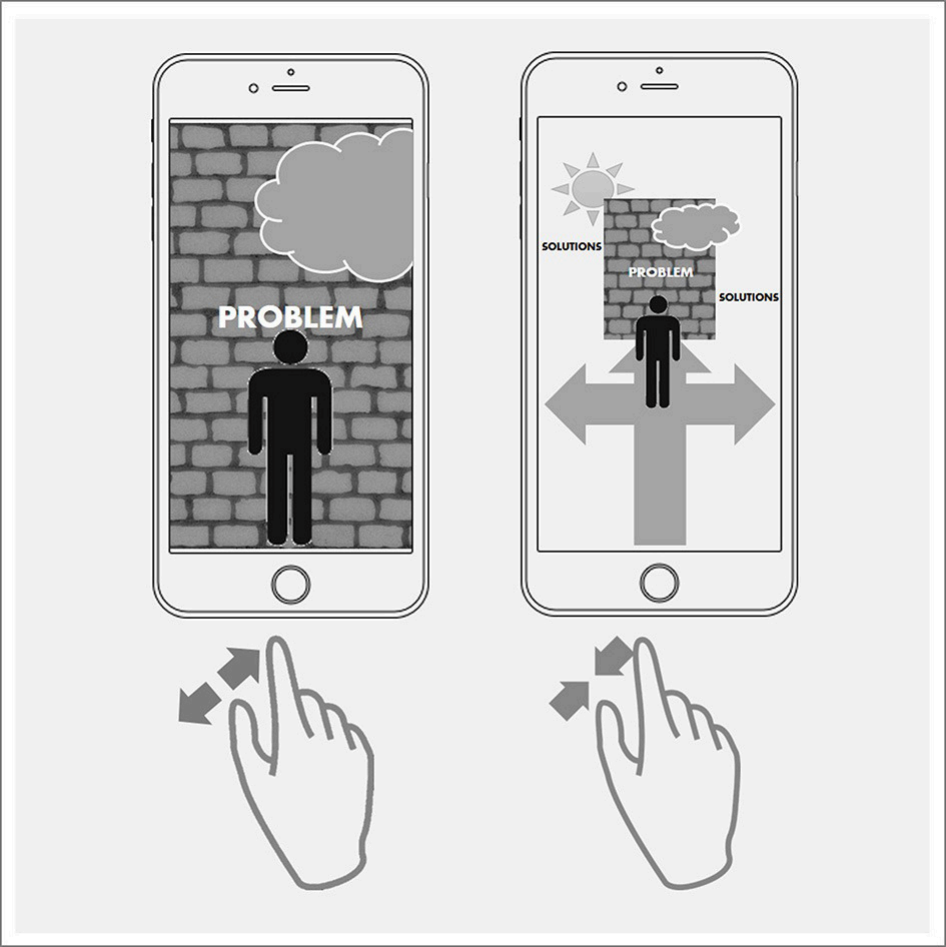
Prototypical visualization of using the zoom gesture to change perspectives on problems and solutions: standing right in front of a problem wall **(Left)** vs. exploring possible ways around the wall **(Right)**.

Besides the advantages of technology to support the experience of change through their ubiquitous presence, technology also offers advanced opportunities for the continuous activation of a positive therapeutic frame on different levels of interaction design. In contrast to face-to-face settings, where the coach repeatedly re-activates supportive perspectives through reflection, exercises and dialogue, interactive technology could do this continuously. Mobile apps, for example, could trigger helpful perspectives and reflections through interface design, menu titles, visualizations, or also interaction attributes. “Psychological weight” (e.g., heavy accuses, heavy problems, high barriers) could be represented through physical weight, realized through the force needed to move elements by touch gestures on a display. Likewise, technology could shift the focus from problems to solutions. For example, zooming in and out of a problem plays with the metaphor of standing right in front of a problem wall, where one sees nothing but the problem and overlooks all the ways around it. While performing the zoom gesture, one can actually experience the opportunities to change the perspective and thereby, experiences the diverse paths of positive change (see Figure 3 for a prototypical visualization). This approach of (here: visually) shifting the focus away from to problems to solutions is in parallel to basic theoretical concepts of solution-focused coaching (e.g., de Shazer et al., 1986; Bamberger, 2011; de Shazer and Dolan, 2012). As already outlined in section Potential, these approaches assume that the focus on the problem, turning in paralyzing circles, does not foster but rather prevents any positive change, often called problem trance or problem hypnosis. Zooming out of the problem and experiencing the power of this more distanced perspective, thus, could be a first step out of the problem trance.

### Strategy 3: unintended success

A third strategy could be to avoid the challenging barrier and arena of change and actually initiate change (=success) without a prior clear intention. The unintended success may provide a full dose of sweetness, a lure for further success. The more conventional path of change, that is, realizing a discrepancy between ideal and real self, committing and trying to change, always includes the risk for failure and self-blame. Fear and negative thoughts may suck energy that is better spent on the actual change. In contrast, the unconventional path of unintended success is virtually risk free. The moment one becomes aware of the change, it was already successful and maybe easier than thought. This provides a safe basis for commitment to further change, since one already knows that change is possible. If the (unintended) change is not successful, it is also not a failure—since one never committed to wanting that change.

The aim is not to nudge people into a direction they would not consider attractive. Instead, the idea of unintended success is to create some positive surprise about oneself. One may hesitate to consider (secret) ideals as explicit goals, simply because the way appears too bitter. However, if for some reason we are pushed into an unexpected challenge, some of these challenges might turn out to be easier to manage than we expect. For example: Friends taking us on a hiking tour turning out much longer than expected, gets us to over-accomplish our aims. Furthermore, a small holiday resort with no cigarette machine nearby effects the smoking behavior and thereby indicates that a smoke-free day is possible. Ordeals as a possibility to detect one's actual skills are also a prominent technique in solution focused coaching (e.g., Bamberger, 2011); also Milton H. Erickson already used ordeals in a therapeutic context (Zeig, 2013). In hindsight, one may even be thankful for being pushed into that challenge and having discovered one's strength and abilities. Unintended success thus provides a basis for *post-hoc* commitment.

Likewise, a healthy nutrition app making meal suggestions each day could skip fish and meat for 1 week. However, only after the week has passed, the user hears “congratulations on your first vegetarian week!” Speaking in terms of Tromp et al. (2011), who differentiate product influence on behavior along the dimensions of force and salience, such an approach could be classified as rather strong but with a (at first) hidden influence. The user is aware of the fact that the app cares about healthy nutrition, but is not aware that this includes also attempts of vegetarian living. After having unintendedly succeeded in having a vegetarian week, the user may commit to a goal such as having a vegetarian day once a week, may decide to become vegetarian, or deliberately decide against it.

Altogether, the strategy of unintended success probably represents the most challenging one, also from an ethical perspective. It may be difficult to decide what kind of “success” actually creates “positive surprise about oneself” and corresponds to the user's personal ideals. There is a high responsibility to navigate between positive support and manipulation. Note, however, that the essence of this strategy is not to trick the user, but just to initiate an action before a critical reflection happens. The strategy aims to create a frame of success on previous actions that allows the user to feel proud, and then opens up the possibility to further engage in the change, if wanted.

## Conclusions

The present work highlights the responsible role of technology as a mediator of well-being and therapeutic interaction and discusses possible ways for a practical integration of psychology and technology design. A particular emphasis is on the bitter-sweet ambivalence of change, including potential relapses and risks of self-threat, so that technology-mediated interventions adapted from (positive) psychology can have a positive impact to full effect.

All people will likely profit from approaching their ideals, but for some of them, bitter components and barriers to change appear more difficult than for others. Especially the former turn out as a relevant target group for self-improvement technologies. Stibe (2016) calls this group “January 1st”: people who would like to change their routines, but rarely succeed in doing so. On the contrary, people with comparatively high levels of motivation and skills for self-improvement are “self-driven people,” and Stibe (2016) even argues that persuasive technologies might become unnecessary for this group. However, many existing behavioral intervention technologies are primarily suited for this non-target group, i.e., people who are already passionate about self-optimization, supporting intentional self-change through reminders and feedback. Those who could profit the most (e.g., insufficiently active people), are highly sensitive to user experience issues and especially hesitant toward technology as a means for behavior change (Yang et al., 2015). Thus, it is primarily important that the design of self-improvement technologies is adjusted to the special needs of the people who actually require support in changing themselves.

The present model aims for anyone to enter the positive change spiral by actively considering bitter and sweet components. Being rooted in the ideas of positive psychology and the belief in people's capability, it assumes that self-initiated change is possible, but further acknowledges that implementing it into daily life is a highly strenuous process. Hence, understanding what makes change more bitter or sweeter for people appears as a key factor for success.

As an interdisciplinary field, positive technology requires a frame to combine best knowledge from different disciplines. The collective task is to translate insights about human behavior and motivation from psychology and the social sciences into design concepts and product-mediated interventions, realized through technology. The present conceptualization of change wants to provide a contribution in this direction. It may function as a frame for the systematic identification of potential to support people during the process of change. It depicts relevant forces and possible strategies in order to support change and possible ways for technology to intervene. The present model is no substitute for the exhaustive study of psychological theory. However, it aims to provide an easy and catchy frame for designers and HCI specialists in the field of positive technology to position their project. Designers can sketch the kind of change they want to support and become aware of relevant psychological mechanisms. As such, the bitter-sweet concept can be a starting point for the definition of general strategies and “therapeutic goals,” which can be then further refined by reference to relevant theory.

## Author contributions

The author confirms being the sole contributor of this work and approved it for publication.

### Conflict of interest statement

The author declares that the research was conducted in the absence of any commercial or financial relationships that could be construed as a potential conflict of interest.

## Appendix

Items to assess bitter and sweet factors of change and principal component analysis (varimax rotation).

**Table:**

**Scale/Item**	**Component**			
**The product …**	**1**	**2**	**3**	**4**
*Bitter: Confrontation with deficits*				
… makes me realize that I have not yet reached my ideal state	0.79			
… confronts me with my dissatisfaction with the current state	0.78			
… shows me that I haven't done enough yet	0.71			
*Bitter: Demand*				
… does not accept that I put off getting active any longer		0.89		
… definitely reminds me to start the planned behavior change		0.82		
… clearly states that it is time to act	0.55	0.53		
*Sweet: Autonomy*				
…leaves it up to me how to design the change process			0.86	
… lets me make autonomous decisions how to proceed			0.88	
… provides considerable freedom about how to reach my goals			0.76	0.32
*Sweet: Encouraging Feedback*				
… praises me for my actions				0.88
… provides positive feedback				0.85
… acknowledges small steps on the way to self-improvement				0.76

*All items were originally in German; component loadings <0.30 are suppressed*.
